# Supplementary material for: A network dynamical simulation model for the study of antibiotic resistance in microbial communities
Source: Gut Microbes. 2026 Sep 19;18(1):2734703. doi: 10.1080/19490976.2026.2734703 (PMC13596919; doi:10.1080/19490976.2026.2734703)
Supplement: Supplementary file 1 [file KGMI_A_2734703_SM9053.zip › supplementary_file_1/Figures/er_delta_gc_size_n300.pdf]

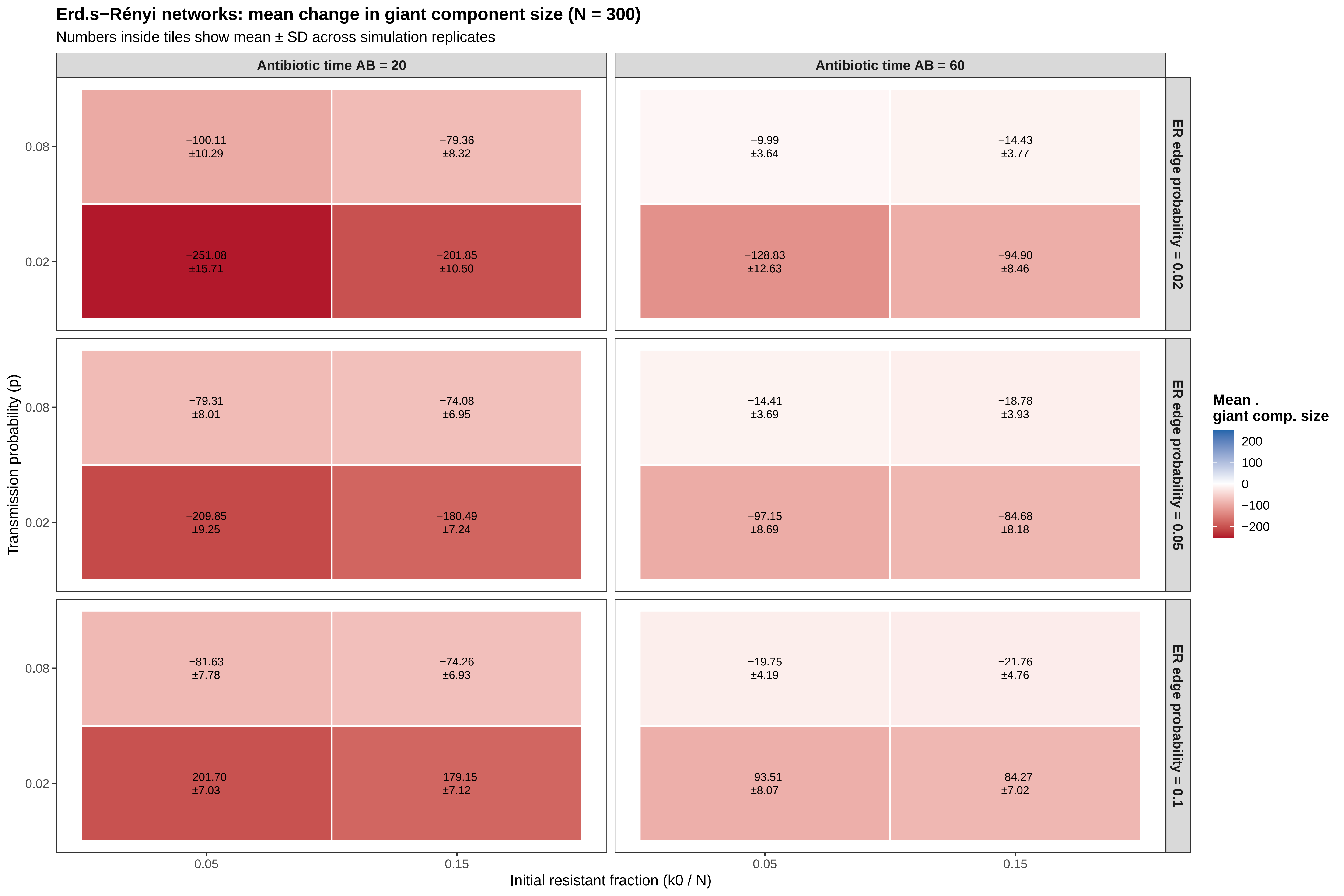

Erd.s−Rényi networks: mean change in giant component size (N = 300)
Numbers inside tiles show mean ± SD across simulation replicates
Antibiotic time AB = 20
Antibiotic time AB = 60
ER edge probability = 0.02
ER edge probability = 0.05
ER edge probability = 0.1
Transmission probability (p)
0.08
0.02
Initial resistant fraction (k0 / N)
0.05
0.15
−100.11 ±10.29
−79.36 ±8.32
−251.08 ±15.71
−201.85 ±10.50
−9.99 ±3.64
−14.43 ±3.77
−128.83 ±12.63
−94.90 ±8.46
−79.31 ±8.01
−74.08 ±6.95
−209.85 ±9.25
−180.49 ±7.24
−14.41 ±3.69
−18.78 ±3.93
−97.15 ±8.69
−84.68 ±8.18
−81.63 ±7.78
−74.26 ±6.93
−201.70 ±7.03
−179.15 ±7.12
−19.75 ±4.19
−21.76 ±4.76
−93.51 ±8.07
−84.27 ±7.02
Mean .
giant comp. size
200
100
0
−100
−200
